# ENHANCING HEALTHCARE VALUE THROUGH INTEROPERABLE E-CARE PLANS: A FRAMEWORK FOR QUALITY IMPROVEMENT

**DOI:** 10.1093/geroni/igae098.4262

**Published:** 2024-12-31

**Authors:** Arlene Bierman, Shari Ling, Johanthan Nebeker

**Affiliations:** Agency for Healthcare Research and Quality, Rockville, Maryland, United States; Centers for Medicare & Medicaid Services, Baltimore, Maryland, United States; Department of Veterans Health Affairs, Washington, District of Columbia, United States

## Abstract

Patient-centered data is critical for providing high-value care and assessing quality including functional and cognitive status, goals, and outcomes that matter most to people; these are often missing in Electronic Health Records (EHRs). Furthermore, lack of interoperability makes it challenging to share and aggregate data across clinicians and care settings, leading to fragmented and/or duplicative care. E-care plans (eCP) and data standards that focus on goal setting and outcome management are in development, and hold promise to support person- centered care planning across sites and settings of care. E-Care Plans support care coordination and quality improvement and inform future healthcare value-focused policies—such as those related to equitable coverage, quality improvement, measurement, payment, and innovation model planning. Interoperable eCPs could be integrated into clinical care and across the healthcare ecosystem. This integration will enhance care coordination and facilitate seamless information exchange. In this session, Federal officials from the CMS, VA, and AHRQ will explore the benefits of eCPs and discuss the work underway to support the implementation of these plans in clinical care. This presentation will also discuss efforts to develop value sets and FHIR mappings to enable bidirectional information exchange and the concurrent pilot testing of applications with patient data. E-care plans have the potential improve patient safety, reduce burden, support the efficient exchange of patient data such as cognitive function, physical function, and individual goals to support improving person-centered outcomes, advancing health equity, conducting economic analysis, and tailored program development.

